# Output-Only Modal Identification of Civil Engineering Structures Based on Parametric Complexity Pursuit

**DOI:** 10.3390/s26082417

**Published:** 2026-04-15

**Authors:** Jianming Li, Jinjin Gao, Shixiang Zhang, Violeta Mircevska, Maosen Cao

**Affiliations:** 1 College of Mechanics and Engineering Science, Hohai University, 8 Fochengxi Road, Nanjing 211100, China; lijianming@hhu.edu.cn (J.L.);; 2 Jiangsu Huatong Engineering Technology Co., Ltd., Nanjing 210017, China; 3 Nanjing Hydraulic Research Institute, 225 Guangzhou Road, Nanjing 210029, China; 4 Institute of Earthquake Engineering and Engineering Seismology, Ss. Cyril and Methodius University in Skopje, Todor Aleksandrov Street, 1000 Skopje, North Macedonia

**Keywords:** output-only modal identification, blind identification, complexity pursuit, Heritage Court Tower, system identification

## Abstract

This paper proposes a novel parametric output-only modal identification method, termed parametric complexity pursuit (PCP), for accurate identification of modal parameters in civil engineering structures. The proposed method extends the complexity pursuit (CP) algorithm through a system representation. Unlike the standard CP approach, which extracts modes individually, the PCP algorithm transforms CP into a parametric formulation that enables global identification of all modal parameters of interest. This parametric framework provides a rigorous theoretical foundation aligned with structural dynamics and explicitly accounts for mutual influences among different modes, thereby enhancing both accuracy and robustness. Numerical experiments on a simulated 3-DOF system under various damping conditions and closely spaced modes demonstrate that PCP maintains consistent identification accuracy across all damping levels, exhibiting true damping-independent performance. In contrast, conventional CP suffers from marked accuracy degradation as damping increases and fails to properly identify the closely spaced modes. Application to the HCT building further confirms the practical effectiveness of PCP, with comparative analysis demonstrating its superiority over existing output-only techniques in modal separation capability and identification accuracy. The proposed PCP method offers a robust and theoretically grounded framework for output-only modal identification, particularly advantageous in challenging scenarios involving high damping and closely spaced modes.

## 1. Introduction

The characterization of linear system dynamics has relied heavily on modal identification techniques developed over recent decades. The identified modal parameters, including natural frequencies, damping ratios and mode shapes, provide overall structural information in applications of structural health monitoring [1,2,3,4,5], model updating [6,7,8], vibration control [9,10], seismic evaluation [11,12], etc. Despite the widespread use of traditional input–output modal identification algorithms [13], their application to civil engineering structures is often limited by the practical challenges of applying measurable artificial excitation. Consequently, research attention has shifted toward output-only modal identification algorithms, which estimate modal parameters solely from vibration response measurements without requiring any knowledge of the input [14]. Instead of relying on artificial excitation, output-only methods leverage ambient excitations such as wind, water flow, microtremors, and traffic that act on the structure under operational conditions. These ambient excitations typically exhibit a broad frequency content, approximating white noise.

Most of the output-only modal identification methods were established based on parametric system models in the form of polynomial or state-space representations, e.g., data-driven/covariance-driven stochastic subspace identification (SSI-DATA/SSI-COV) [15], polyreference least-squares complex frequency-domain methods (PolyMAX) [16], auto-regression moving average vector (ARMAV) [17], natural excitation technique (NExT) [18]. The existing parametric methods are characterized by high precision owing to the rationality of parametric system models [19]. However, they normally have difficulty determining the system order and input parameters, for which applying the stabilization diagram requires vast expert experience and computing resources [20]. It is more difficult to guarantee the validity of these methods when applied to large-scare infrastructures with weak or non-stationary excitation. In contrast, non-parametric modal identification methods, e.g., frequency domain decomposition (FDD) [21], peak-picking (PP) [22], Hilbert–Huang transform (HHT) [23], share the benefits of simple implementation and high computational efficiency, yet their precision is relatively low due to the absence of a parametric mathematical model and their weak theoretical connection to structural dynamics.

Lately, blind source separation (BSS), originally applied as a signal processing tool, has been attracting much attention from the structural vibration community [24]. BSS aims to separate observed signals into underlying source signals with limited or no knowledge of the source characteristics. The reason why BSS works well in modal identification is that it can form a relationship with the mode superposition principle in structural dynamics theory [25].

The most popular BSS algorithms dealing with modal identification are second-order blind identification (SOBI) [26,27], independent component analysis (ICA) [28] and algorithm for multiple unknown signal extraction (AMUSE) [29]. The ICA algorithm assumes that the observed data are composed of independent components, namely statistically independent sources. The independent assumption is tenable in many fields but proved to be too restrictive for modal identification and thus the ICA-based methods are limited to undamped or lightly damped cases. In addition, the ICA algorithm involves high-order statistics and complicated optimization computation, the problems of convergence and precision are increased for modal identification applications. The SOBI algorithm makes a weaker assumption of uncorrelated sources and focuses on second-order statistics supported by matrix diagonalization. However, sources estimated by SOBI might include information from other modes [30]. The SOBI-based methods are considered to be under-performing in cases of non-proportional damping and closely spaced modes [26].

In recent years, a novel BSS strategy termed complexity pursuit (CP) has shown great potential in output-only modal identification [31]. The CP algorithm seeks for sources that have maximal temporal predictability instead of following imposed statistical assumptions [32]. It explicitly integrates both statistical and temporal information of the observed signals, and thus finds special utility in recovering modal responses with temporal structures. The learning rule of the CP algorithm lies in the observation that physically realizable sources are basically generated by the motion of mass [33], which is well suitable for describing the vibration of structures. Compared with the existing BSS-based methods, the CP-based method displays the superiority in dealing with the highly damped modes and closely spaced modes with little adjusted parameters [31]. It is also shown to be robust against contaminating noise. However, as with the existing BSS-based methods and other non-parametric methods, the precision of the CP-based method is restricted by the absence of the parametric system model.

Inspired by a parametrizing framework which the authors have recently developed [34,35,36], the present paper proposes a novel parametric output-only modal identification method based on complexity pursuit, termed parametric complexity pursuit (PCP). Unlike the standard CP-based method, which extracts local modal information mode by mode using single-degree-of-freedom (SDOF) fitting techniques, the proposed PCP method derives a discrete-time state-space model that serves as the parametric system representation. This transforms CP into a parametric formulation, enabling global identification of all interested modal parameters. The proposed PCP algorithm offers a rigorous theoretical foundation that aligns with the underlying structural dynamics and explicitly accounts for mutual influences among different modes, consequently enhancing both accuracy and robustness. Extensive numerical experiments were conducted on a simulated 3-DOF system to validate the performance of the PCP algorithm under both proportional and non-proportional damping conditions with various damping ratios, as well as in the presence of closely spaced modes. To further demonstrate its practical applicability, the proposed algorithm is applied to the modal identification of the Heritage Court Tower building. The identified results are compared with those obtained from existing state-of-the-art output-only modal identification methods, confirming the effectiveness of the proposed method.

## 2. Extracting Modal Responses via Complexity Pursuit

### 2.1. Blind Source Separation for Modal Identification

BSS attempts to extract componential sources embedded in the observed signals. For the application of modal identification, the observed signals are structural dynamic responses, which can be acceleration, velocity, displacement or strain of structures depending on the sensor type. The relation between sources and observed signals can be expressed by linear instantaneous mixing model [37] as:

(1) y(t)=As(t)

where $y(t)={\left[y_{1}(t),\cdots ,y_{l}(t)\right]}^{T}\in {\mathbb{R}}^{l\times 1}$ is the vector of observed signals at time t; $s(t)={\left[s_{1}(t),\cdots ,s_{m}(t)\right]}^{T}\in {\mathbb{R}}^{m\times 1}$ is the vector of sources at time t; and $A\in {\mathbb{R}}^{l\times m}$ is the unknown mixing matrix. The following de-mixing model is then considered

(2) s(t)=Wy(t)

where $W={\left[w_{1}^{T},\cdots ,w_{m}^{T}\right]}^{T}\in {\mathbb{R}}^{m\times l}$ is the matrix of m de-mixing vectors. Obviously, W is the (generalized) inverse of A. The generalized inverse of A existing requires that l≥m, corresponding to the overdetermined or deterministic problem. For the underdetermined scenario (l<m), the observed signals can be filtered into distinct frequency ranges, thereby allowing the corresponding modal responses and modal matrix for each range to be solved independently. The task of BSS is to find a de-mixing matrix W such that each de-mixing vector $w_{j}$ can extract a signal $\varsigma_{j}(t)$ as an scaled estimate of $s_{j}(t)$. $\varsigma_{j}(t)$ is given by:

(3) $$\varsigma_{j}(t)=w_{j}y(t)$$

It is fairly evident that BSS has practical utility for output-only modal identification. In structural dynamics, the vibration responses x(t) are composed of the modal responses q(t) through modal expansion as:

(4) y(t)=φq(t)

where φ is the modal matrix consisting of mode shapes corresponding to the measured DOFs, whereas the modal responses q(t) contain information on natural frequencies and damping ratios. The similarity between Equations (1) and (4) leads to the application of BSS to the modal identification problem. The de-mixing model can be expressed as:

(5) $$q(t)=\varphi^{-1}y(t)$$

Note that φ is a real matrix only if the proportional damping condition is satisfied, which is adaptable for lightly damped linear structures. Following the application of BSS algorithms, the modal responses q(t) and modal matrix φ can be estimated by q(t)≈s(t) and $\varphi \approx W^{-1}$.

### 2.2. Measuring Signal Complexity

The CP algorithm seeks for the de-mixing vector $w_{j}$ by exploiting the signal complexity, which is rarely involved in the existing BSS algorithms. Hyvärinen described the complexity as an augmented property in ICA framework [38]. However, the measure of complexity is strictly derived from information theory, which is generally too complicated to be used in real applications. Stone introduced a simpler measure of complexity formulated in terms of temporal predictability [32]. The measure of temporal predictability *F* can be defined as:

(6) $$F(\varsigma_{j})=\log\frac{\sum_{t=1}^{T}{\left({\bar{\varsigma }}_{j}(t)-\varsigma_{j}(t)\right)}^{2}}{\sum_{t=1}^{T}{\left({\tilde{\varsigma }}_{j}(t)-\varsigma_{j}(t)\right)}^{2}}$$

The temporal predictability F measures the “overall variance” and “temporal roughness” of the extracted signal by reflecting the level to which $\varsigma_{j}(t)$ can be predicted by the long-term moving average ${\bar{\varsigma }}_{j}(t)$ and short-term moving average ${\tilde{\varsigma }}_{j}(t)$, respectively. ${\bar{\varsigma }}_{j}(t)$ and ${\tilde{\varsigma }}_{j}(t)$ are formed by exponentially weighted moving sum of signals measured up to t−1, given by:

(7) $${\bar{\varsigma }}_{j}(t)=\lambda_{L}{\bar{\varsigma }}_{j}(t-1)+(1-\lambda_{L})\varsigma_{j}(t-1),\;{\tilde{\varsigma }}_{j}(t)=\lambda_{S}{\tilde{\varsigma }}_{j}(t-1)+(1-\lambda_{S})\varsigma_{j}(t-1)$$

The weightings are defined as:

(8) $$\lambda_{L/S}=2^{-1/h_{L/S}},\;0\leq \lambda_{L/S}\leq 1$$

where the half-life $h_{L}$ corresponding to $\lambda_{L}$ is larger than $h_{S}$ corresponding to $\lambda_{S}$, typically $h_{L}=900000$, $\lambda_{S}=1$ [32]. From Equation (6), it follows that the signal predictability F includes both statistical and temporal terms. It is particularly suited for extracting time-structured signals like modal responses.

Substituting Equation (3) into Equation (8) yields:

(9) $$F(\varsigma_{j})=F(w_{j},y)=\log\frac{w_{i}\bar{R}w_{i}^{T}}{w_{i}\tilde{R}w_{i}^{T}}$$

where $\bar{R}$ and $\tilde{R}$ are the long-term and short-term covariance matrices between observed signals x(t), respectively. The elements of the matrices are expressed as:

(10) $${\bar{r}}_{ij}=\sum_{t=1}^{T}\left(y_{i}(t)-{\bar{y}}_{i}(t))(y_{j}(t)-{\bar{y}}_{j}(t)\right),\;{\tilde{r}}_{ij}=\sum_{t=1}^{T}\left(y_{i}(t)-{\tilde{y}}_{i}(t))(y_{j}(t)-{\tilde{y}}_{j}(t)\right)$$

### 2.3. Maximizing Temporal Predictability

For a given observation dataset y, the learning rule of the CP algorithm is to minimize the signal complexity, which means to seek for the de-mixing vector $w_{j}$ that maximizes the temporal predictability $F(w_{j},y)$.

The derivative of $F(w_{j},y)$ in Equation (9) with respect to $w_{j}$ yields:

(11) $$\nabla_{w_{j}}F=\frac{2w_{j}}{\sum_{t=1}^{T}{\left({\bar{\varsigma }}_{j}(t)-\varsigma_{j}(t)\right)}^{2}}\bar{R}-\frac{2w_{j}}{\sum_{t=1}^{T}{\left({\tilde{\varsigma }}_{j}(t)-\varsigma_{j}(t)\right)}^{2}}\tilde{R}$$

The maximum of the temporal predictability F can be located by iteratively updating the de-mixing vector $w_{j}$ as:

(12) $$w_{j}=w_{j}+\eta \nabla_{w_{j}}F$$

where η is the learning rate, typically η=0.001.

It is obvious that the global maximum of F is guaranteed to be found by gradient ascent. When the gradient of F from Equation (11) equals to zero, it obtains:

(13) $$w_{j}\bar{R}=\frac{\sum_{t=1}^{T}{\left({\bar{\varsigma }}_{j}(t)-\varsigma_{j}(t)\right)}^{2}}{\sum_{t=1}^{T}{\left({\tilde{\varsigma }}_{j}(t)-\varsigma_{j}(t)\right)}^{2}}w_{j}\tilde{R}$$

The values of $w_{j}$ that satisfy Equation (13) yield extrema in F, which is considered as a generalized eigenvalue problem. $w_{j}$ can then be solved as eigenvectors of the matrix ${\tilde{R}}^{-1}\bar{R}$. The corresponding eigenvalues are $\gamma_{j}=\sum_{t=1}^{T}{\left({\bar{\varsigma }}_{j}(t)-\varsigma_{j}(t)\right)}^{2}/\sum_{t=1}^{T}{\left({\tilde{\varsigma }}_{j}(t)-\varsigma_{j}(t)\right)}^{2}$. In this way, the de-mixing matrix W and sources s(t) are obtained simultaneously. The modal responses q(t) can be estimated accordingly.

To mitigate edge effects in the optimization process arising from sudden data segment boundaries, the observed signals can be windowed before applying complexity pursuit. A Gaussian window is employed herein to attenuate the signal amplitude at the boundaries. The window function is defined as:

(14) $$w_{g}(t)=e^{-\mu^{2}{\left(2t/T-1\right)}^{2}/2}$$

where μ is the spectral bandwidth parameter, which controls the width of the Gaussian window, typically μ=2.5 [26]. A larger μ leads to increased spectral bandwidth but reduced temporal window width.

## 3. Parameterizing Strategy for Modal Parameter Estimation

### 3.1. Parametric System Model

After estimating the modal responses q(t) and modal matrix φ as described in Section 2.3, the mode shapes can be obtained immediately from the columns of φ. Normally, the natural frequencies and damping ratios are calculated from q(t) mode by mode through SDOF fitting [39]. As discussed, the accuracy of the estimated modal parameters is limited due to the absence of the parametric system model and the neglect of the interactions between different modes. A parametric system model is derived herein to parameterize the CP-based modal identification process, thereby enhancing the accuracy.

For a N DOF structural system with proportional damping, the vibration responses can be described by the following second-order equation of motion

(15) $$M\ddot{u}(t)+C\dot{u}(t)+Ku(t)=f(t)$$

where u(t), $f(t)\in {\mathbb{R}}^{N\times 1}$ are the vector of displacement and excitation force at time t; M, C, $K\in {\mathbb{R}}^{N\times N}$ are the mass, damping, and stiffness matrices, which are real symmetric matrices.

It can be inferred from the eigenvalue problem related to Equation (15) that

(16) $$M\Phi \Lambda^{2}+C\Phi \Lambda +K\Phi =0$$

where $\Phi \in {\mathbb{R}}^{N\times N}$ satisfies φ=LΦ, $L\in {\mathbb{R}}^{l\times N}$ is the permutation matrix used for selecting the measured DOFs; $\Lambda =\mathrm{diag}\{[\lambda_{1},\cdots ,\lambda_{N}]\}\in {\mathbb{C}}^{N\times N}$; $\lambda_{j}=-\xi_{j}\omega_{j}+i\omega_{j}\sqrt{1-\xi_{j}^{2}}$; $\xi_{j}$ is the damping ratio corresponding to the j th mode; $\omega_{j}$ is the circular frequency corresponding to the j th mode; i is the imaginary unit, $i^{2}=-1$; and diag{•} forms a diagonal matrix.

By adding the identity $M\dot{u}(t)=M\dot{u}(t)$, Equation (15) can be reformulated as the following first-order state-space equation [40]:

(17) $$\begin{array}{l} \dot{x}(t)=A_{c}x(t)+B_{c}f(t)\\ y(t)=C_{c}x(t)+D_{c}f(t) \end{array}$$

where $x(t)={\left[\dot{u}{\left(t\right)}^{T},u{\left(t\right)}^{T}\right]}^{T}\in {\mathbb{R}}^{2N\times 1}$ is the state vector; $A_{c}\in {\mathbb{R}}^{2N\times 2N}$, $B_{c}\in {\mathbb{R}}^{2N\times N}$, $C_{c}\in {\mathbb{R}}^{l\times 2N}$, $D_{c}\in {\mathbb{R}}^{l\times 2N}$ are the system, input influence, output and direct transform matrices. It is assumed that measurements are collected by accelerometers, the above matrices are given by:

(18) $$A_{c}=\left[\begin{array}{cc} -M^{-1}C & -M^{-1}K\\ I & 0 \end{array}\right],\;B_{c}=\left[\begin{array}{c} M^{-1}\\ 0 \end{array}\right],\;C_{c}=-LM^{-1}\left[\begin{array}{cc} C & K \end{array}\right],\;D_{c}=LM^{-1}$$

Consider the following similarity transformation

(19) x(t)=Ψz(t)

where Ψ, $\Lambda_{c}\in {\mathbb{C}}^{2N\times 2N}$ are given by:

(20) $$\Psi =\left[\begin{array}{cc} \Phi \Lambda & \Phi \Lambda^{*}\\ \Phi & \Phi \end{array}\right],\;\Lambda_{c}=\left[\begin{array}{cc} \Lambda & 0\\ 0 & \Lambda^{*} \end{array}\right]$$

$z(t)\in {\mathbb{C}}^{2N\times 1}$ can be written as $z(t)={\left[g{\left(t\right)}^{T},g{\left(t\right)}^{H}\right]}^{T}$. Substituting Equations (16) and (19) into Equation (17) yields:

(21) $$\begin{array}{l} \dot{z}(t)=\Lambda_{c}z(t)+L_{c}f(t)\\ y(t)=E_{c}z(t)+D_{c}f(t) \end{array}$$

where $L_{c}\in {\mathbb{C}}^{2N\times 2N}$, $E_{c}\in {\mathbb{C}}^{l\times 2N}$ are given by:

(22) $$L_{c}=\Psi^{-1}B_{c},\;E_{c}=C_{c}\Psi =L\Phi \left[\begin{array}{cc} \Lambda^{2} & \Lambda^{*2} \end{array}\right]$$

Substituting Equation (4) into Equation (21) yields:

(23) $$\begin{array}{l} \dot{z}(t)=\Lambda_{c}z(t)+L_{c}f(t)\\ q(t)=V_{c}z(t)+G_{c}f(t) \end{array}$$

where $V_{c}\in {\mathbb{C}}^{N\times 2N}$ and $G_{c}\in {\mathbb{R}}^{N\times 2N}$ are given by:

(24) $$V_{c}=\varphi^{-1}C_{c}\Psi ,\;G_{c}={\left(\Phi M\right)}^{-1}$$

Note that if displacement or velocity is measured, the similar form of the equation can be derived.

It is seen from Equation (23) that the vector of modal responses q(t) instead of observed signals y(t) are served as the output vector and can be estimated by the CP algorithm as described in Section 2. Equation (23) can be used as a parametric system model which makes it possible to parameterize the modal identification procedure.

The system model Equation (23) is represented in continuous time. It needs to be converted to discrete time for adjustment of the outputs to measurements. Also consider that the system is under ambient excitation; the corresponding discrete time model is:

(25) $$\begin{array}{l} z_{k+1}=\Lambda_{d}z_{k}+\mu_{k}\\ q_{k}=V_{d}z_{k}+\nu_{k} \end{array}$$

where $z_{k}=z(k\Delta t)$; $q_{k}=q(k\Delta t)$; $\mu_{k}\in {\mathbb{R}}^{2N\times 1}$ and $\nu_{k}\in {\mathbb{R}}^{N\times 1}$ are the input and output noise, $\Lambda_{d}\in {\mathbb{C}}^{2N\times 2N}$ and $V_{d}\in {\mathbb{C}}^{N\times 2N}$ are given by:

(26) $$\Lambda_{d}=\left[\begin{array}{cc} e^{\Lambda \Delta t} & 0\\ 0 & e^{\Lambda^{*}\Delta t} \end{array}\right],\;V_{d}=V_{c}$$

### 3.2. MDOF Modal Parameter Estimation

On the basis of the parametric system described in Equation (25), the natural frequencies $f_{j}=\omega_{j}/2\pi$ and damping ratios $\xi_{j}$ can be obtained through an MDOF estimation approach similar to SSI-COV as follows.

Calculating the time-lagged covariance matrices R(τ) of $q_{k}$ and assembling in a block Toeplitz matrix $T\in {\mathbb{R}}^{Ni\times Ni}$ as:

(27) $$T=\left[\begin{array}{cccc} R(i) & R(i-1) & \cdots & R(1)\\ R(i+1) & R(i) & \cdots & R(2)\\ \vdots & \vdots & \ddots & \vdots \\ R(2i-1) & R(2i-2) & \cdots & R(i) \end{array}\right]$$

where $R(k)=E\{q_{k}q_{k}^{T}\}$.

Applying singular value decomposition (SVD) to T yields:

(28) $$T=\left[\begin{array}{cc} U_{1} & U_{0} \end{array}\right]\left[\begin{array}{cc} S_{1} & 0\\ 0 & S_{0} \end{array}\right]\left[\begin{array}{c} V_{1}^{T}\\ V_{0}^{T} \end{array}\right]\approx U_{1}S_{1}V_{1}^{T}$$

where $U_{1}$, $V_{1}\in {\mathbb{R}}^{Ni\times 2N}$ are orthogonal matrices; $S_{1}\in {\mathbb{R}}_{+}^{2N\times 2N}$ is a diagonal matrix of singular values in descending order.

According to the state-space theory, the block Toeplitz matrix T has the following factorization property:

(29) $$T=O\Gamma =\left[\begin{array}{c} V_{d}\\ V_{d}\Lambda_{d}\\ \vdots \\ V_{d}\Lambda_{d}^{i-1} \end{array}\right]\left[\Lambda_{d}^{i-1}\begin{array}{cccc} G & \cdots & \Lambda_{d}G & G \end{array}\right]$$

where $O\in {\mathbb{C}}^{Ni\times 2N}$ is the observability matrix; $\Gamma \in {\mathbb{C}}^{2N\times Ni}$ is the reversed controllability matrix. By comparing Equations (28) and (29), O and Γ are calculated by:

(30) O=U1S112, Γ=S112V1T

Matrix $\Lambda_{d}$ can then be computed by exploiting the shift invariance property of O and conducting the eigenvalue decomposition as:

(31) $$\Lambda_{d}=P^{-1}O{\left(1:N(i-1),:\right)}^{†}O(N+1:Ni,:)P$$

where $P\in {\mathbb{C}}^{2N\times 2N}$ is the matrix of eigenvectors.

Finally, the identified natural frequencies ${\hat{f}}_{j}$ and damping ratios ${\hat{\xi }}_{j}$ are obtained from $\Lambda_{d}$ as:

(32) f^j=12πRe(λj)2+Im(λj)2, ξ^j=−Re(λj)2πfj

The matrix of identified mode shapes $\hat{\varphi }$ can be estimated as:

(33) $$\hat{\varphi }=W^{-1}O(1:N,:)P$$

## 4. Numerical Experiments: 3-DOF Mass-Spring-Dashpot System

### 4.1. Proportional Damping

**Figure 1:**
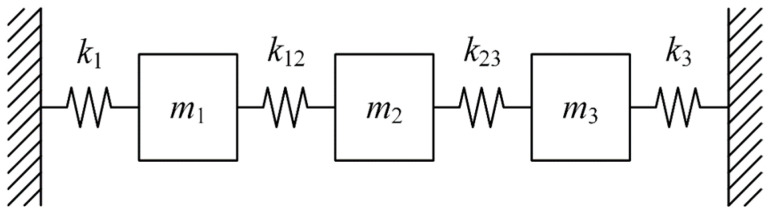
The 3-DOF mass-spring-dashpot system.

In order to validate the proposed PCP modal identification method, numerical simulations through 3-DOF mass-spring-dashpot systems (Figure 1) were conducted in this section. Three masses are connected by springs with both ends fixed. Free vibration with initial displacement $y(0)={\left[\begin{array}{ccc} 0 & 1 & 0 \end{array}\right]}^{T}$ and initial velocity $\dot{y}(0)={\left[\begin{array}{ccc} 0 & 0 & 1 \end{array}\right]}^{T}$ are included. The Newmark-Beta algorithm was utilized to generate the dataset of displacement responses sampled at 10 Hz with the time length of 200 s.

The long-term and short-term half-life parameters $h_{L}$ and $h_{S}$ in Equation (7) were set to 900,000 and 1, respectively, which have been shown to yield stable and reliable performance. The number of block rows i in Equation (27) was set to 20. The spectral bandwidth parameter μ in Equation (14) was set to 2.5. The superiority of PCP is demonstrated through comparison with the standard CP method using SDOF frequency domain fitting [41] to estimate modal parameters of each mode. The accuracy of identified natural frequencies and damping ratios is assessed using the mean relative error (MRE) as:

(34) $$\mathrm{MRE}(\hat{f},f)=\frac{1}{N}\sum_{j=1}^{N}\frac{\left|{\hat{f}}_{j}-f_{j}\right|}{f_{j}}\times 100\%,\;\mathrm{MRE}(\hat{\xi },\xi )=\frac{1}{N}\sum_{j=1}^{N}\frac{\left|{\hat{\xi }}_{j}-\xi_{j}\right|}{\xi_{j}}\times 100\%$$

The correlation between the identified and true mode shapes are evaluated by introducing the modal assurance criterion (MAC), defined as:

(35) $$\mathrm{MAC}({\hat{\varphi }}_{j},\varphi_{j})=\frac{{\left({\hat{\varphi }}_{j}^{T}\varphi_{j}\right)}^{2}}{\left({\hat{\varphi }}_{j}^{T}{\hat{\varphi }}_{j})(\varphi_{j}^{T}\varphi_{j}\right)}\times 100\%$$

The closer the MAC is to 100%, the higher the identification accuracy of mode shapes.

For comparative purposes, the physical parameters of the 3-DOF system are consistent with those in refs. [28,31]. The mass matrix M, stiffness matrix K, and damping matrix C of the system are:

(36) $$M=\left[\begin{array}{ccc} 2 & 0 & 0\\ 0 & 1 & 0\\ 0 & 0 & 3 \end{array}\right],\;K=\left[\begin{array}{ccc} 2 & -1 & 0\\ -1 & 1 & -1\\ 0 & -1 & 2 \end{array}\right],\;C=\alpha M$$

**Figure 2:**
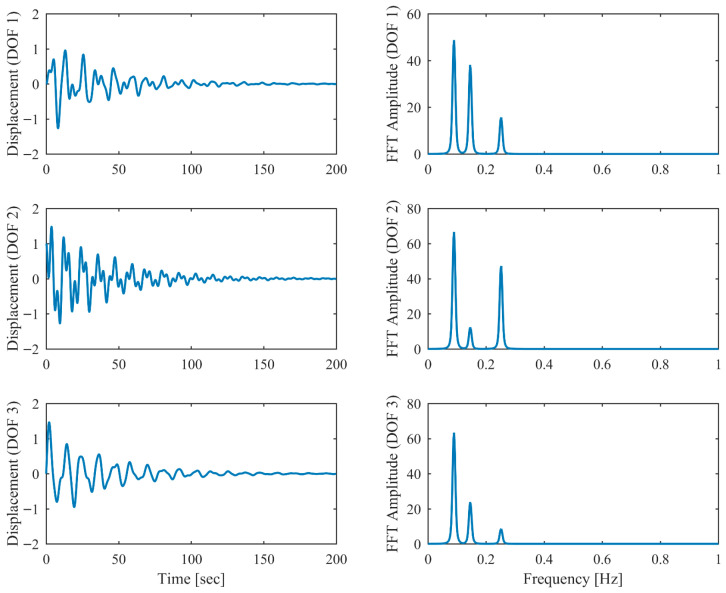
The displacement responses in proportional damping (α = 0.05) in free vibration.

**Figure 3:**
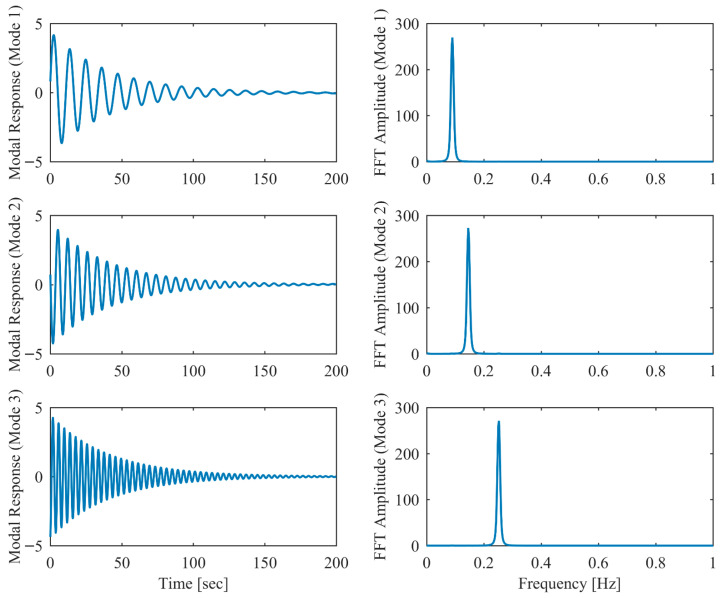
The modal responses separated by PCP in proportional damping (α = 0.05) in free vibration.

**Figure 4:**
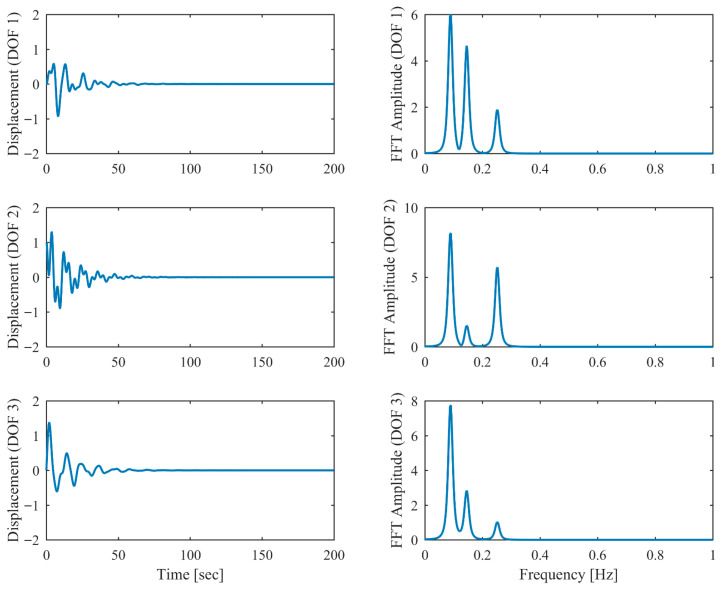
The displacement responses in proportional damping (α = 0.13) in free vibration.

**Figure 5:**
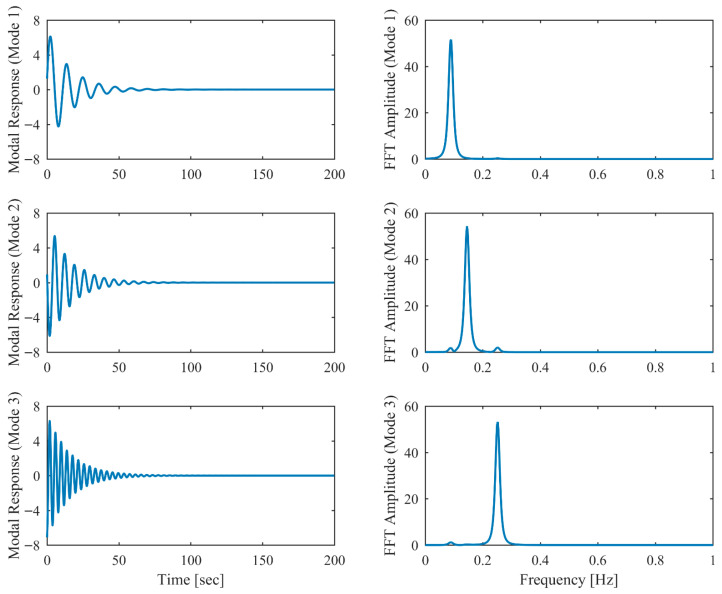
The modal responses separated by PCP in proportional damping (α = 0.13) in free vibration.

**Figure 6:**
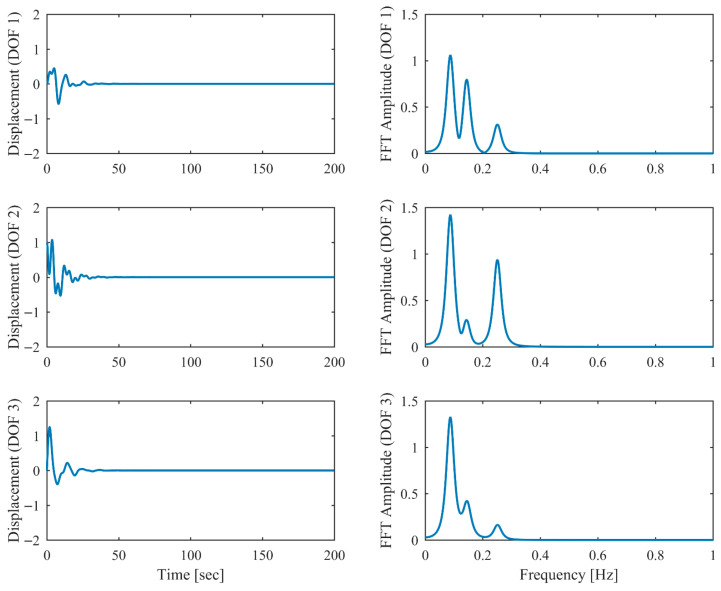
The displacement responses in proportional damping (α = 0.25) in free vibration.

**Figure 7:**
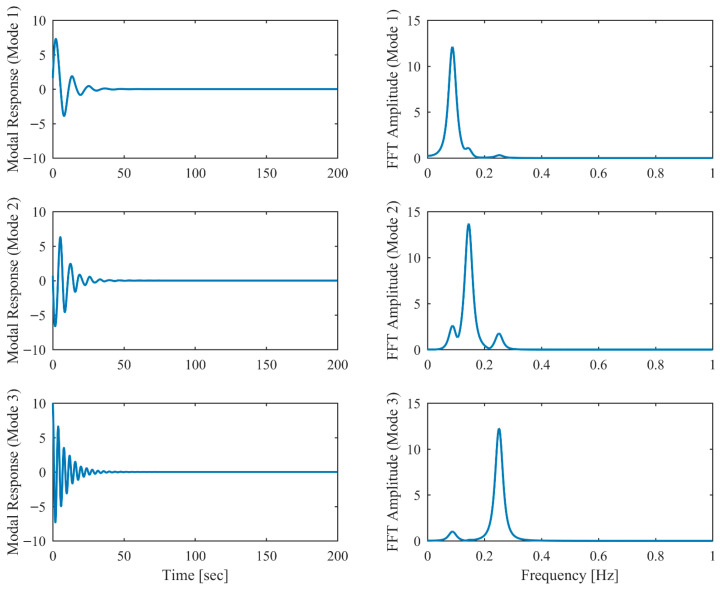
The modal responses separated by PCP in proportional damping (α = 0.25) in free vibration.

Proportional damping with different levels (α = 0.05, 0.13 and 0.25) is considered first. The results of the modal parameters estimated by PCP and CP, along with the theoretical values, are listed in Table 1 and Table 2, with identification accuracy quantified through MRE and MAC. Intriguingly, the MRE of natural frequencies and damping ratios with respect to PCP stabilize at around 0.1% and 0.2%, respectively, regardless of the damping levels. As α varies from 0.05 to 0.25, the MRE of natural frequencies with respect to PCP increases from 0.2% to 1.6% and the MRE of damping ratios grows from 1.7% to 4.3%, indicating that the identification accuracy of PCP maintains consistency across different damping levels, while CP exhibits significant accuracy degradation at high damping levels. Overall, the proposed PCP method achieves superior accuracy and enhanced robustness over the standard CP method in identifying natural frequencies and damping ratios. As evidenced by the MAC values in Table 2, both PCP and CP demonstrate high accuracy in mode shape identification, with PCP exhibiting marginally better performance especially when damping is high. The displacement responses and modal responses separated by PCP in both time and frequency domains with different damping levels are depicted in Figure 2, Figure 3, Figure 4, Figure 5, Figure 6 and Figure 7. As shown in Figure 3, the system responses are distinctly separated into monotonic modal responses with concentrated peaks when damping is low. In comparison, Figure 7 exhibits a faster decay of oscillations and flattened peaks with reduced amplitude due to rapid energy dissipation at high damping levels. The system responses are not fully separated when damping is high, which might affect the accuracy of SDOF fitting utilized in the standard CP method. However, as evidenced by Table 1 and Table 2, the insufficient modal separation has negligible impact on the identification results of PCP, owing to its MDOF modal parameter estimation procedure that inherently accounts for intermodal relationships.

### 4.2. Non-Proportional Damping

The previous example was slightly modified to investigate the more general non-proportional damping conditions. The mass matrix and stiffness matrix are preserved from the preceding discussion, whereas the damping matrix is modified by adding a non-diagonal term as:

(37) $$\begin{array}{l} C=\alpha M+C_{n}\\ C_{n}=\left[\begin{array}{ccc} 0.00 & -0.02 & 0.02\\ -0.02 & 0.00 & 0.03\\ 0.02 & 0.03 & 0.00 \end{array}\right] \end{array}$$

where α retains its previously specified values of 0.5, 0.13 and 0.25. Evidently, complex modes exist in the non-proportional case [42].

As in previous analysis, the modal parameter results identified by PCP and CP as well as the true values are listed in Table 3 and Table 4, with identification accuracy quantified through MRE and MAC. It can be seen from Table 3 that the MREs with respect to different α remain approximately consistent with the previous example in proportional condition. The results suggest that the proposed PCP method achieves satisfactory accuracy and outperforms the CP method even though the proportional assumption is slightly violated, which may be attributed to the superior compatibility inherent in the proposed system model. For the mode shape identification results listed in Table 4, PCP exhibits a modest advantage over CP, especially at high damping levels, maintaining consistency with the proportional damping cases. Overall, the PCP demonstrates damping-independent performance, representing a significant advantage over CP whose performance degrades under high damping conditions. The modal responses separated by PCP exhibit close resemblance to proportional damping cases, and therefore are not presented in this context. Similarly, this derivation has negligible influence on the identification results of PCP.

**Figure 8:**
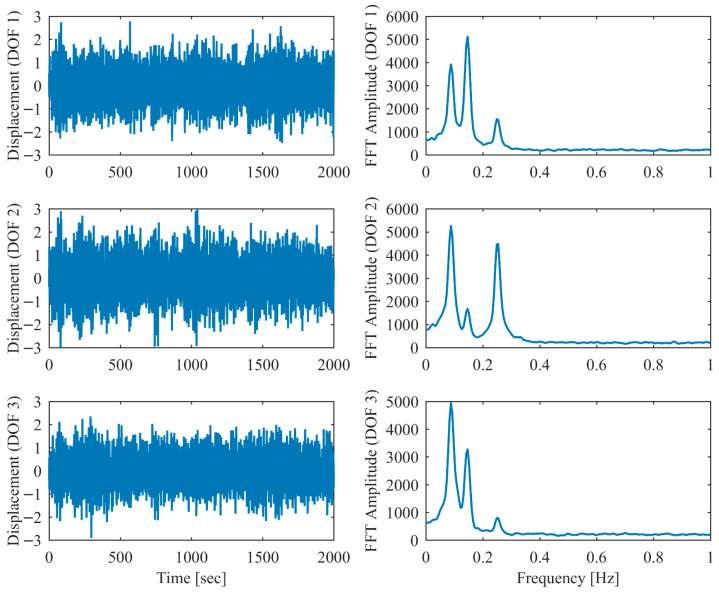
The displacement responses in proportional damping (α = 0.05) in ambient vibration.

**Figure 9:**
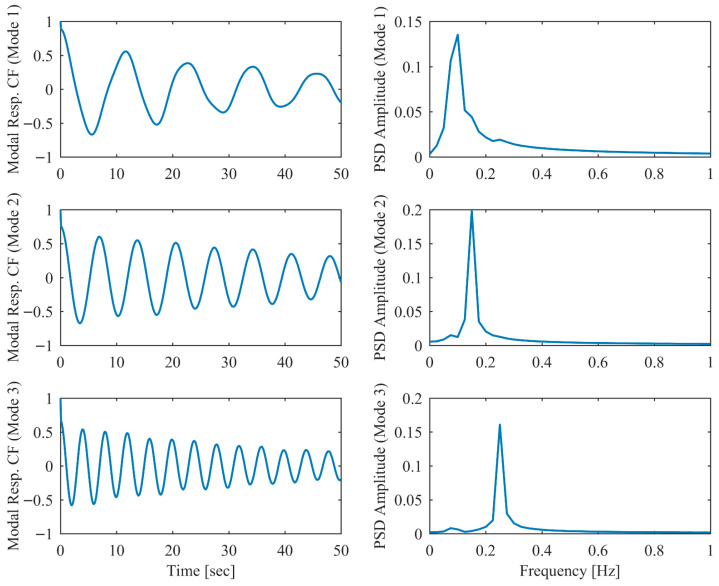
The modal responses separated by PCP in proportional damping (α = 0.05) in ambient vibration.

**Figure 10:**
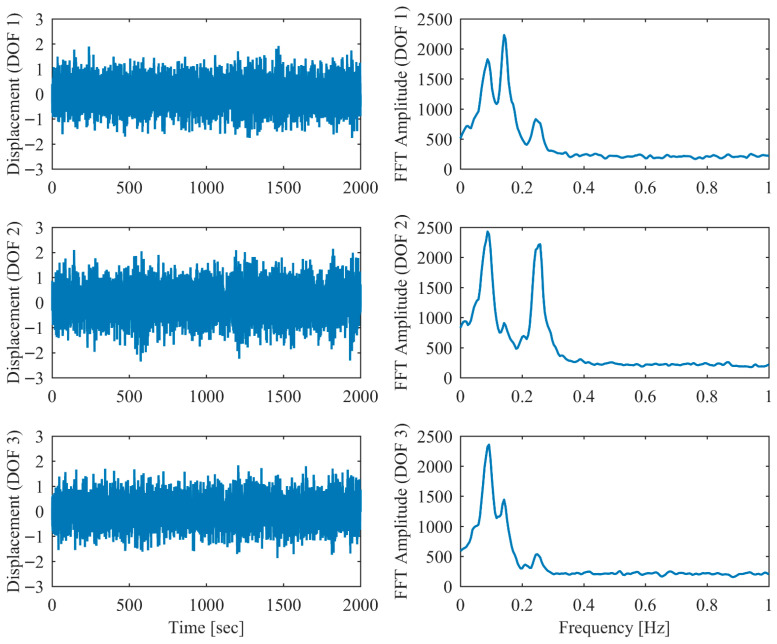
The displacement responses in proportional damping (α = 0.13) in ambient vibration.

**Figure 11:**
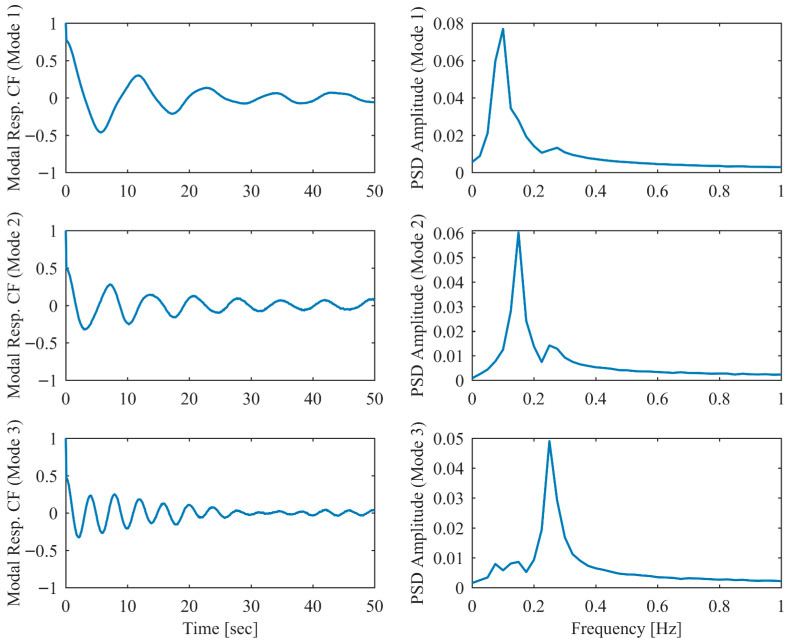
The modal responses separated by PCP in proportional damping (α = 0.13) in ambient vibration.

**Figure 12:**
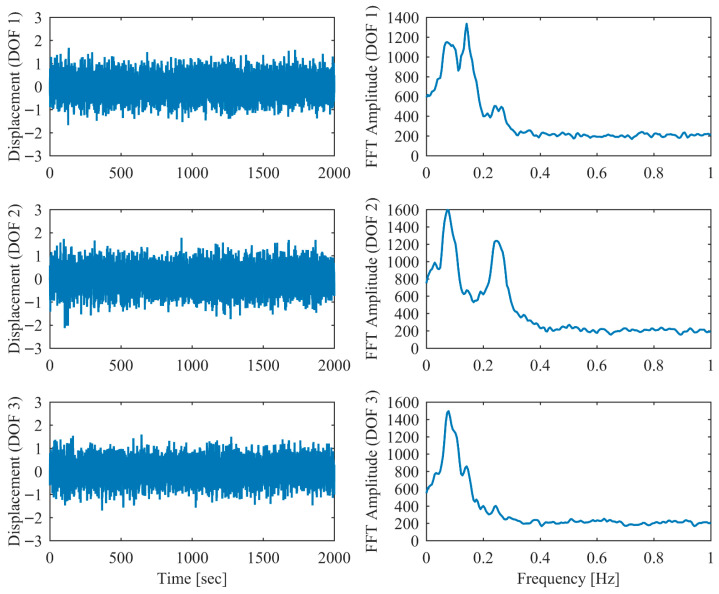
The displacement responses in proportional damping (α = 0.25) in ambient vibration.

**Figure 13:**
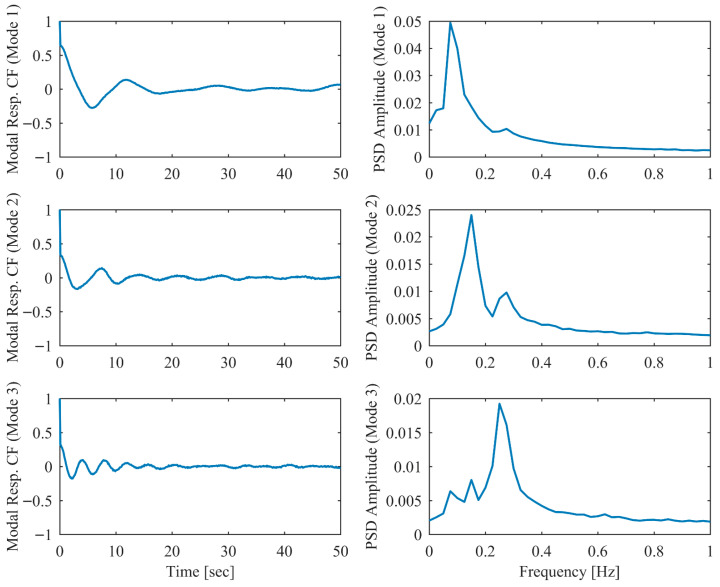
The modal responses separated by PCP in proportional damping (α = 0.25) in ambient vibration.

In order to validate the robustness of the proposed PCP method under more realistic ambient vibration conditions, the non-proportional damping example subjected to random excitation was investigated. White Gaussian noise was applied as the excitation source at each DOF. Additionally, measurement noise with a signal-to-noise ratio of 10 dB was introduced into the displacement responses. The modal parameter results identified by PCP and CP as well as the true values are listed in Table 5. It can be seen that the PCP method consistently demonstrated higher identification accuracy compared to the conventional CP method. Figure 8, Figure 9, Figure 10, Figure 11, Figure 12 and Figure 13 depict the displacement responses in both the time and frequency domains, along with the autocorrelation functions (CFs) and auto-power spectral density functions (PDFs) of the modal responses separated by PCP under varying damping levels. These results demonstrate that the PCP method possesses a robust capacity for modal response separation.

### 4.3. Closely Spaced Modes

In order to evaluate the robustness of the proposed PCP method against closely spaced modes, the model with proportional damping in Section 4.1 was slightly modified as follows

(38) $$M=\left[\begin{array}{ccc} 1 & 0 & 0\\ 0 & 5 & 0\\ 0 & 0 & 1 \end{array}\right],\;K=\left[\begin{array}{ccc} 2 & -1 & 0\\ -1 & 1 & -1\\ 0 & -1 & 2 \end{array}\right],\;C=\alpha M$$

where α = 0.5, 0.13 and 0.25, respectively, as previously defined.

**Figure 14:**
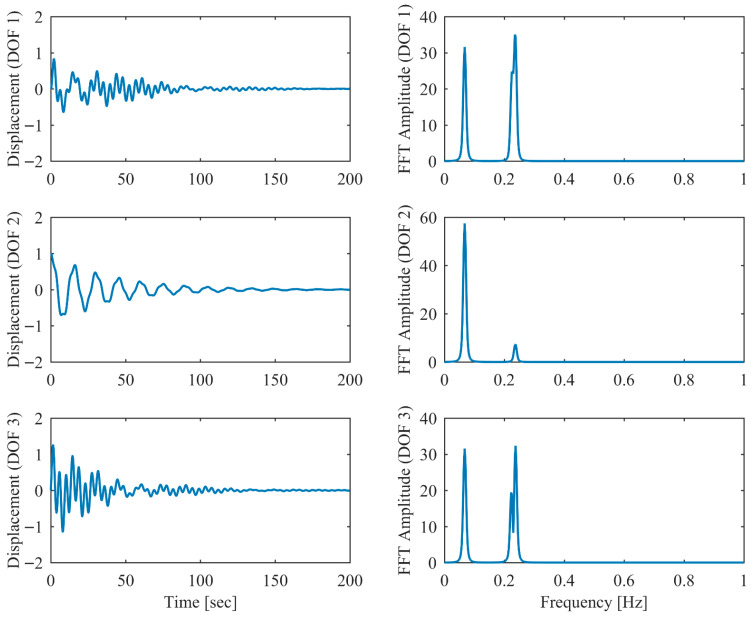
The displacement responses in proportional damping with closely spaced modes (α = 0.05) in free vibration.

**Figure 15:**
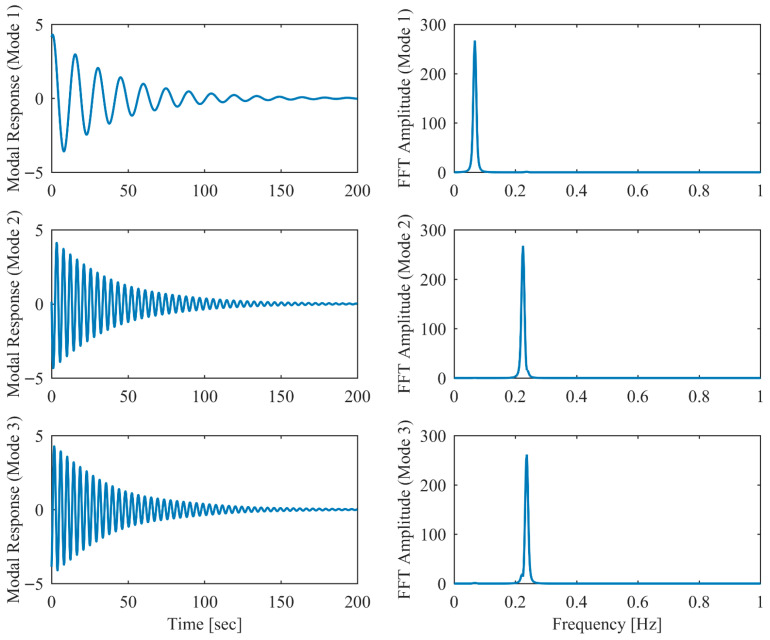
The modal responses separated by PCP in proportional damping with closely spaced modes (α = 0.05) in free vibration.

**Figure 16:**
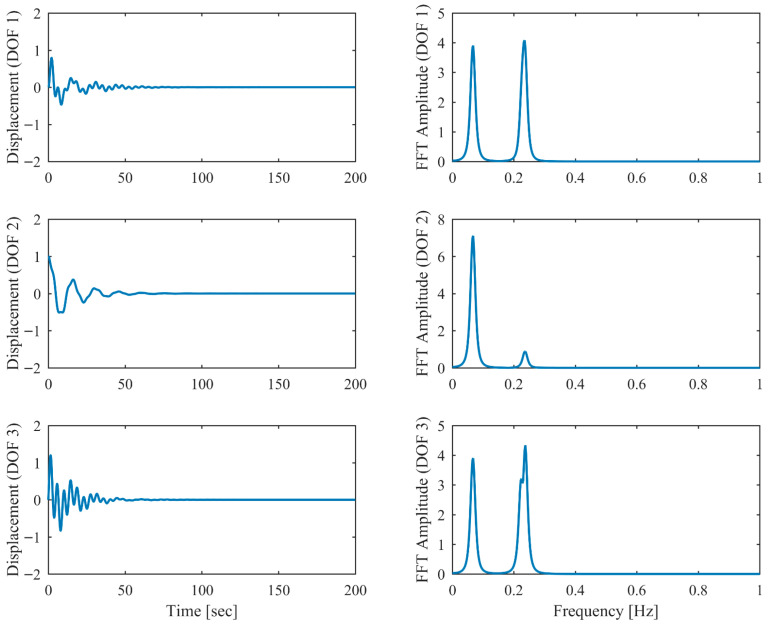
The displacement responses in proportional damping with closely spaced modes (α = 0.13) in free vibration.

**Figure 17:**
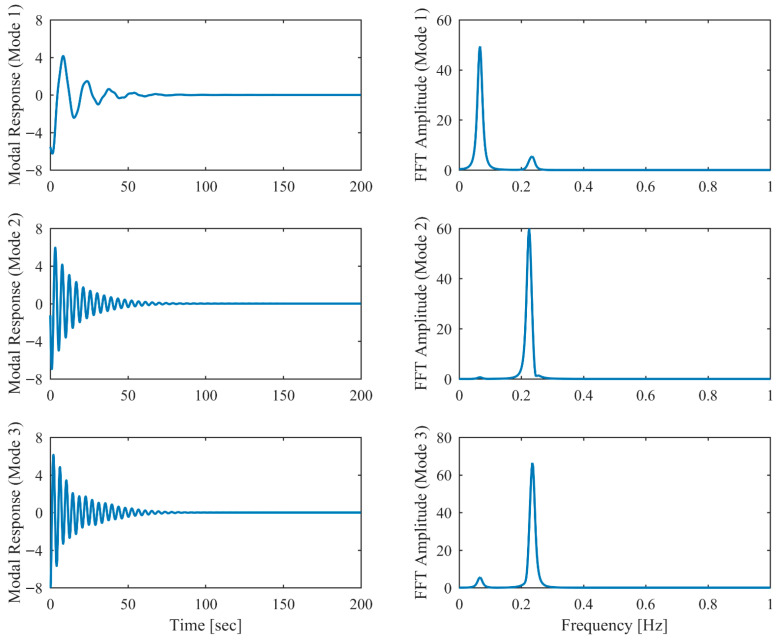
The modal responses separated by PCP in proportional damping with closely spaced modes (α = 0.13) in free vibration.

**Figure 18:**
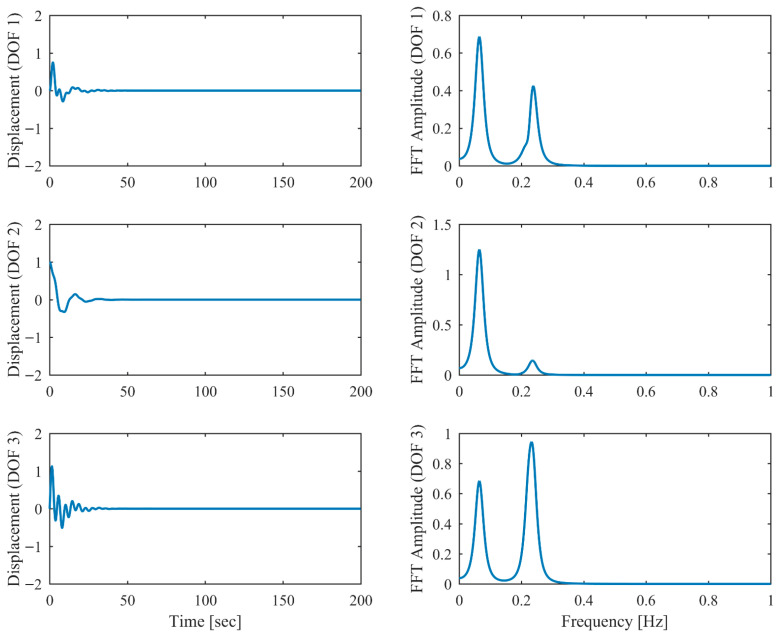
The displacement responses in proportional damping with closely spaced modes (α = 0.25) in free vibration.

**Figure 19:**
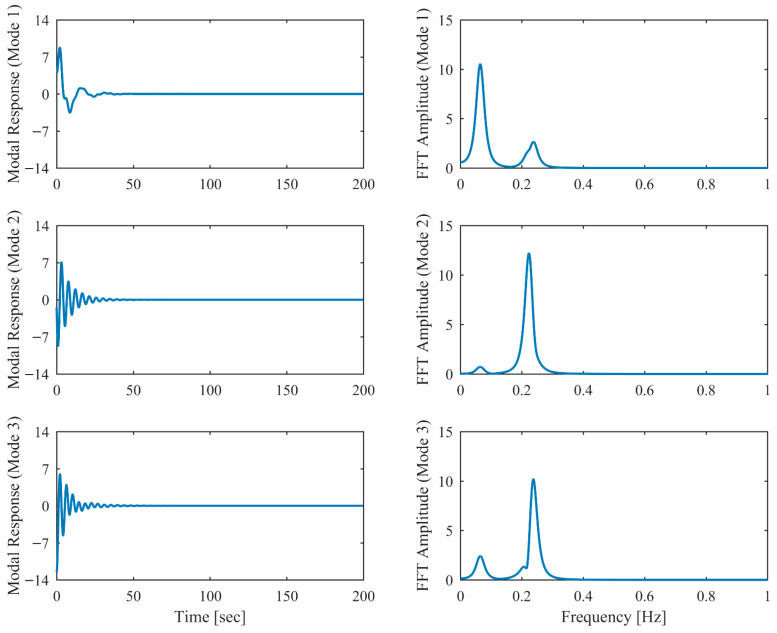
The modal responses separated by PCP in proportional damping with closely spaced modes (α = 0.25) in free vibration.

Table 6 presents the PCP and CP identification results in free vibration with true values, including MRE between estimated and theoretical values. Clearly, the second and third modes are closely spaced, with a mere 5% relative frequency difference. Comparison between Table 1 and Table 6 reveals that the accuracy of the proposed PCP method is less affected by closely spaced modes for both natural frequency and damping estimation, whereas CP exhibits more significant performance degradation in the presence of closely spaced modes. Table 7 lists the MAC between the identified and theoretical mode shapes in free vibration. Clearly, PCP maintains consistently high accuracy in mode shape identification regardless of damping level increases. In contrast, CP demonstrates acceptable accuracy in mode shape identification under low damping conditions, but its performance degrades with increasing damping levels, successively failing to properly identify the second and third modes. The displacement responses and identified modal responses in time and frequency domains with different damping levels are presented in Figure 14, Figure 15, Figure 16, Figure 17, Figure 18 and Figure 19. Clearly, the spectral peaks with respect to the second and third modes in Figure 14, Figure 16 and Figure 18 exhibit significant overlap especially when damping is high. As observed in Figure 15, Figure 17 and Figure 19, even though modal responses are effectively separated when α = 0.5, PCP fails to fully decouple modal responses of different modes when α = 0.13 and 0.25. Nevertheless, this limitation is conclusively overcome by PCP as evidenced by Table 6 and Table 7.

## 5. Real-World Application: Heritage Court Tower Building

**Figure 20:**
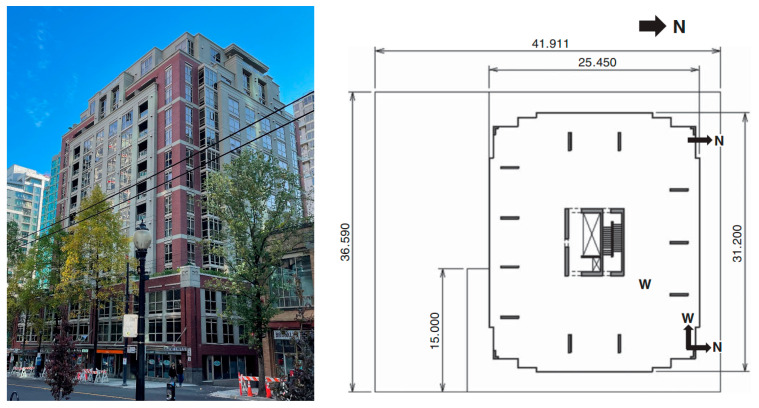
The photograph and typical floor plan of the HCT building.

The proposed PCP method was applied to the Heritage Court Tower (HCT) building, a 15-story reinforced concrete shear core building in downtown Vancouver, British Columbia, Canada. A photograph and typical floor plan of the building are presented in Figure 20. The building is predominantly rectangular in shape, incorporating minor setbacks and projections. The building tower has a compact form, with height-to-width aspect ratios of approximately 1:7 in the east–west direction and 1:3 in the north–south direction. Typical story height is 2.7 m, with the first story elevated to 4.7 m. The standard floor dimensions are approximately 25 × 31 m for upper levels, 36 × 30 m for lower levels, and 42 × 36 m for underground floors. The structural system integrates the elevators and staircases into a central core, forming the main lateral force-resisting system against wind and earthquake actions. The structure consists of an above-ground tower and a three-story underground reinforced concrete parking garage. The garage extends south of the tower while being flush with the tower’s first floor on the remaining three sides, thereby forming an L-shaped podium.

**Figure 21:**
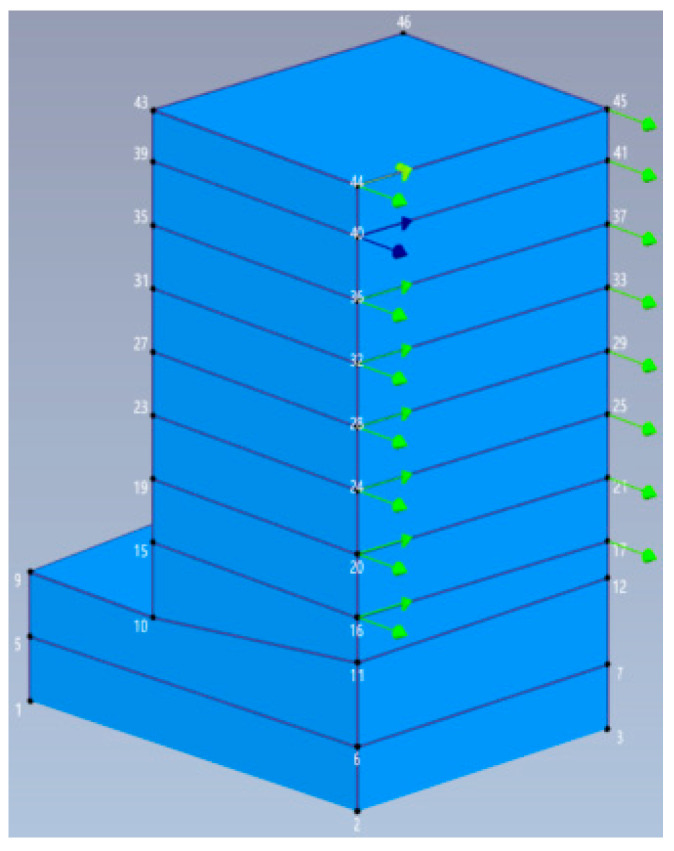
The accelerometer locations and directions in HCT building during the vibration test.

The building was tested under ambient excitation with four data sets. The measurement system comprised eight force-balanced accelerometers (Kinemetrics Inc., Pasadena, CA, USA, Model FBA-11), a signal conditioner (Kinemetrics Inc., Pasadena, CA, USA, M-3), an A/D converter (Keithley Instruments Inc., Solon, OH, USA, Model 575 equipped with an AMM2 broard), and a computer running a customized program AVDA. Six of these accelerometers were deployed as roving sensors on every other floor from the roof down to the second floor, while the remaining two served as fixed reference sensors on the 14th floor. The accelerometers were typically positioned at the northwest and northeast corners of the building, recording responses in two orthogonal directions. The locations and directions of test setups are illustrated in Table 8 and Figure 21. The letter ‘R’ denotes a reference sensor, ‘N’ and ‘W’ represent the northward and westward directions, respectively, and the numerical value in the middle indicates the sensor location number. More details of the test can be found in ref. [43].

**Figure 22:**
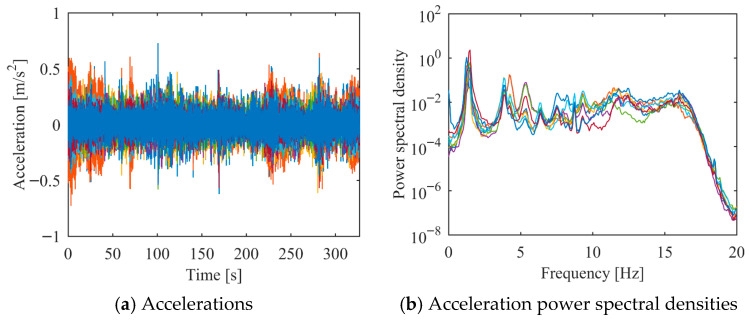
This accelerations and acceleration power spectral densities of the third test setup of the HCT building.

**Figure 23:**
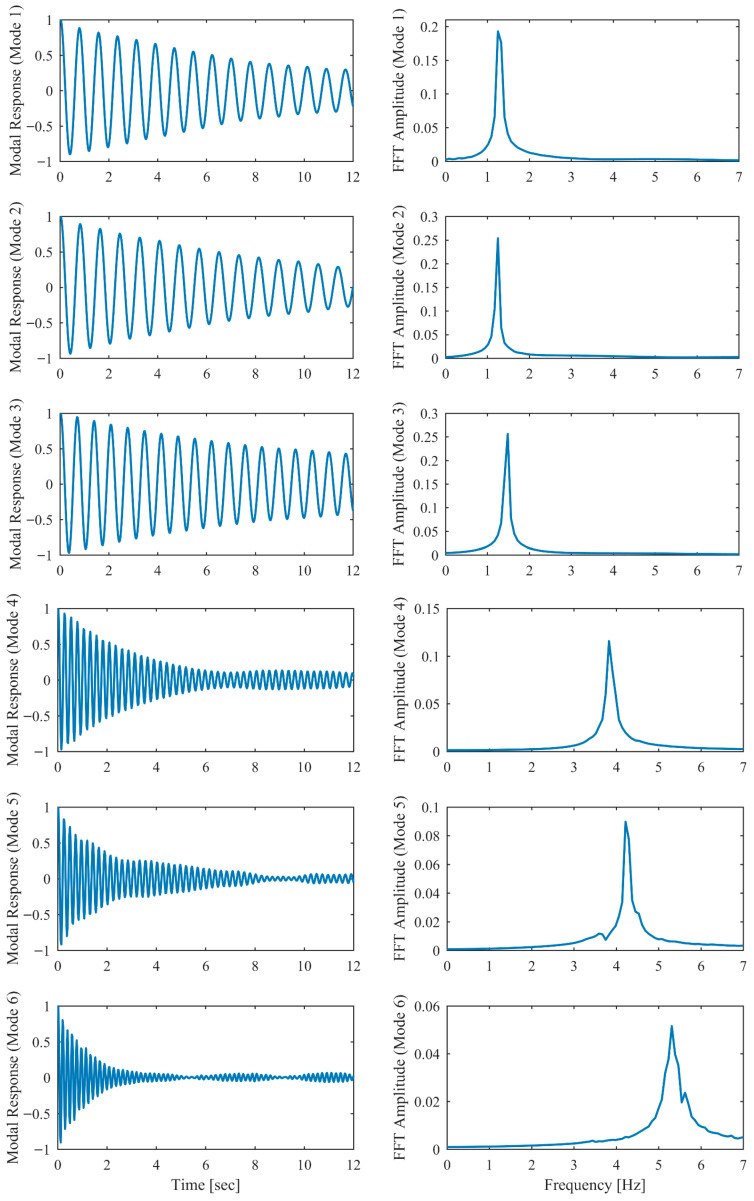
The modal responses of the HCT building separated by PCP.

**Figure 24:**
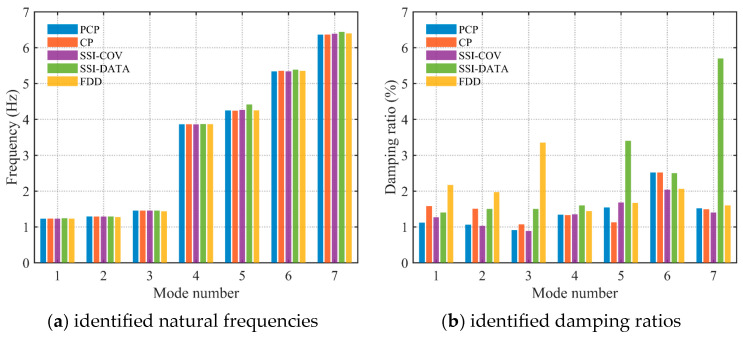
Comparison of natural frequencies and damping ratios identified by different methods.

The third setup was analyzed using the proposed PCP method. The dataset consists of 327 s of acceleration time series, decimated to a Nyquist frequency of 20 Hz. The acceleration responses in both time and frequency domains are plotted in Figure 22. The analysis focuses on the frequency range of 0.0–7.5 Hz. The predefined parameters in the proposed PCP method are configured as follows. The long-term and short-term half-life parameters $h_{L}$ and $h_{S}$ in Equation (7) were assigned values of 900,000 and 1, respectively. The number of block rows i in Equation (27) was set to 100. The spectral bandwidth parameter μ in Equation (14) was taken as 2.5. The first seven modal responses separated by PCP in both time and frequency domains are depicted in Figure 23. The autocorrelation functions of the modal responses demonstrate a clean and monotonically decaying behavior. The frequency content manifests as concentrated and distinct spectral peaks, indicating that the proposed PCP method effectively separates the identified modes. The natural frequencies and damping ratios associated with the first seven modes identified by PCP are presented in Table 9. The results obtained by CP and SSI-COV are also included, along with SSI-DATA and FDD reported in refs. [43,44]. The comparison of natural frequencies and damping ratios associated with the first seven modes identified by different methods are shown in Figure 24. The results obtained by the proposed PCP method are generally consistent with those from other methods, particularly for natural frequencies. Furthermore, the PCP method effectively addresses the challenge of identifying the first three closely spaced modes clustered around 1.25 Hz. These results demonstrate the effectiveness of the proposed PCP method.

**Figure 25:**
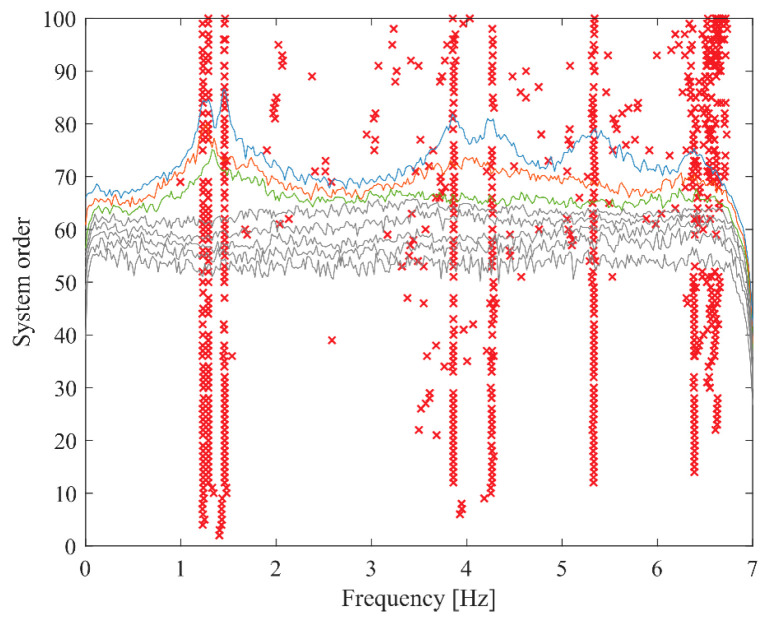
SSI-COV stabilization diagram of the HCT building for the third test setup.

To demonstrate the superiority of the proposed method, the stabilization diagram obtained from SSI-COV along with the singular value spectra from FDD are presented in Figure 25. In the plot, red crosses (×) denote the stable poles identified by SSI-COV, while the colored lines represent the singular value spectra estimated by FDD. While well-separated structural modes exhibit almost stable vertical alignments that correspond with distinct spectral peaks, the proliferation of spurious mathematical poles at elevated system orders heavily obscures the identification process. As a result, it relies heavily on heuristic user intervention and specialized domain expertise to accurately calibrate system orders, interpret complex stabilization behaviors, and effectively discriminate physical structural modes from algorithmic artifacts and noise components. In the identification of FDD, modal peaks are frequently contaminated by spurious spectral contents. The accurate extraction of physical modes, especially closely spaced modes like the first two of the HCT building, demands substantial expert judgment. Consequently, this empirical evidence underscores that despite their theoretical maturity, traditional SSI-COV and FDD algorithms possess significant operational limitations.

**Figure 26:**
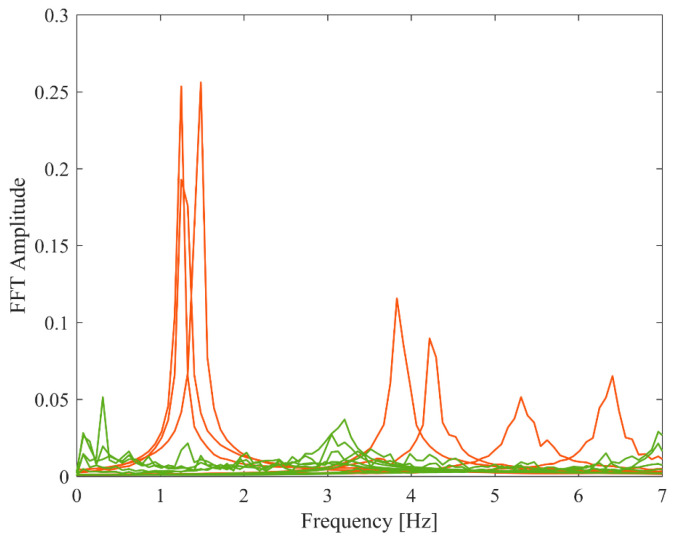
The auto-power spectral density functions of all modal responses separated by PCP.

In contrast, Figure 26 presents the auto-power spectral density functions of all modal responses separated by PCP. The seven physical modes (red lines) and spurious modes (green lines) can be readily distinguished through visual inspection or clustering-based techniques [34]. The proposed PCP method leverages the intuitive nature of the CP method to discern physical modes and determine the system order through inspecting the modal response features, thereby circumventing the need for stabilization diagrams and eliminating the reliance on extensive empirical judgment. This represents a significant reduction in subjectivity compared to stabilization diagram-based methods. Subsequently, by incorporating parametric modeling, the method significantly enhances identification accuracy compared to standard CP method.

## 6. Conclusions

This study proposes a novel parametric output-only modal identification method, termed parametric complexity pursuit (PCP), which extends the CP algorithm through a parametric formulation. By introducing a discrete-time state-space model as the parametric system representation, the proposed PCP algorithm addresses the inherent limitations of the CP-based modal identification and enables global identification of all modal parameters of interest.

The effectiveness and robustness of the PCP algorithm were validated through extensive numerical experiments on a simulated 3-DOF system under both proportional and non-proportional damping conditions with varying damping ratios. The results reveal that PCP maintains consistent modal identification accuracy across all damping levels, exhibiting true damping-independent performance. In contrast, the CP algorithm suffers from marked accuracy degradation as damping increases, highlighting the superior robustness of the PCP algorithm.

Further validation under closely spaced mode conditions reveals that PCP consistently delivers accurate identification of natural frequencies, damping ratios, and mode shapes, whereas CP fails to properly identify higher modes as damping increases. This underscores the superior capability of PCP to effectively handle modal coupling inherent in closely spaced modes.

The practical applicability of the proposed method was further demonstrated through its successful application to the HCT building. Comparative analysis with existing output-only modal identification techniques confirms the robustness of PCP in terms of modal separation capability and modal parameter identification accuracy. The algorithm also exhibits robust performance in identifying closely spaced modes.

Overall, the proposed PCP algorithm provides a rigorous and theoretically grounded framework for output-only modal identification, offering significant advantages over conventional approaches in terms of accuracy, robustness, and adaptability to challenging identification scenarios involving high damping and closely spaced modes.

## Figures and Tables

**Table 1:** The results of natural frequencies and damping ratios in proportional damping cases in free vibration.

α	Method	Natural Frequency (Hz)				Damping Ratio (%)			
		1st Mode	2nd Mode	3rd Mode	MRE (%)	1st Mode	2nd Mode	3rd Mode	MRE (%)
0.05	TRUE	0.0895	0.1458	0.2522	/	4.444	2.730	1.578	/
	PCP	0.0895	0.1457	0.2517	0.1017	4.440	2.726	1.571	0.209
	CP	0.0892	0.1455	0.2518	0.2275	4.561	2.697	1.558	1.696
0.13	TRUE	0.0895	0.1458	0.2522	/	11.554	7.098	4.102	/
	PCP	0.0895	0.1457	0.2517	0.1015	11.544	7.086	4.084	0.220
	CP	0.0886	0.1450	0.2520	0.5471	11.880	6.915	4.045	2.255
0.25	TRUE	0.0895	0.1458	0.2522	/	22.219	13.649	7.888	/
	PCP	0.0895	0.1457	0.2517	0.1005	22.198	13.627	7.855	0.224
	CP	0.0873	0.1429	0.2527	1.5450	23.369	12.826	7.745	4.338

**Table 2:** The results of modal assurance criterion in proportional damping cases in free vibration.

α	Method	Modal Assurance Criterion (%)		
		1st Mode	2nd Mode	3rd Mode
0.05	PCP	99.99999	99.99999	99.99999
	CP	99.99997	99.99996	99.99960
0.13	PCP	99.99999	99.99999	99.99999
	CP	99.97388	99.99564	99.85624
0.25	PCP	99.99999	99.99999	99.99999
	CP	99.39564	98.56429	98.46932

**Table 3:** The results of natural frequencies and damping ratios in non-proportional damping cases in free vibration.

α	Method	Natural Frequency (Hz)				Damping Ratio (%)			
		1st Mode	2nd Mode	3rd Mode	MRE (%)	1st Mode	2nd Mode	3rd Mode	MRE (%)
0.05	TRUE	0.0895	0.1458	0.2522	/	5.478	1.896	1.692	/
	PCP	0.0895	0.1457	0.2517	0.1018	5.473	1.897	1.685	0.185
	CP	0.0892	0.1456	0.2518	0.2432	5.614	1.869	1.673	1.687
0.13	TRUE	0.0895	0.1458	0.2522	/	12.588	6.264	4.216	/
	PCP	0.0895	0.1457	0.2517	0.1015	12.577	6.254	4.198	0.220
	CP	0.0884	0.1452	0.2520	0.5721	12.919	6.096	4.161	2.210
0.25	TRUE	0.0895	0.1458	0.2522	/	23.253	12.817	8.002	/
	PCP	0.0895	0.1457	0.2517	0.1006	23.231	12.796	7.968	0.225
	CP	0.0870	0.1439	0.2526	1.4055	24.440	11.649	7.857	5.342

**Table 4:** The results of modal assurance criterion in non-proportional damping cases in free vibration.

α	Method	Modal Assurance Criterion (%)		
		1st Mode	2nd Mode	3rd Mode
0.05	PCP	99.99999	99.99999	99.99999
	CP	99.99988	99.99999	99.99937
0.13	PCP	99.99998	99.99996	99.99999
	CP	99.96384	99.99814	99.83886
0.25	PCP	99.99988	99.99977	99.99994
	CP	99.34854	98.85924	98.42200

**Table 5:** The results of natural frequencies and damping ratios in non-proportional damping cases in ambient vibration.

α	Method	Natural Frequency (Hz)				Damping Ratio (%)			
		1st Mode	2nd Mode	3rd Mode	MRE (%)	1st Mode	2nd Mode	3rd Mode	MRE (%)
0.05	TRUE	0.0895	0.1458	0.2522	/	5.478	1.896	1.692	/
	PCP	0.0897	0.1458	0.2526	0.1138	5.514	1.902	1.702	0.523
	CP	0.0890	0.1464	0.2517	0.4135	5.836	2.551	1.795	15.729
0.13	TRUE	0.0895	0.1458	0.2522	/	12.588	6.264	4.216	/
	PCP	0.0895	0.1455	0.2526	0.1362	12.581	6.264	4.160	0.462
	CP	0.0907	0.1444	0.2530	0.8756	14.049	6.668	4.909	11.498
0.25	TRUE	0.0895	0.1458	0.2522	/	23.253	12.817	8.002	/
	PCP	0.0895	0.1453	0.2519	0.1715	23.167	12.868	7.995	0.286
	CP	0.0886	0.1448	0.2576	1.2713	23.858	14.031	9.650	10.895

**Table 6:** The results of natural frequencies and damping ratios in closely spaced modes cases in free vibration.

α	Method	Natural Frequency (Hz)				Damping Ratio (%)			
		1st Mode	2nd Mode	3rd Mode	MRE (%)	1st Mode	2nd Mode	3rd Mode	MRE (%)
0.05	TRUE	0.0676	0.2251	0.2371	/	5.889	1.768	1.678	/
	PCP	0.0676	0.2247	0.2367	0.1217	5.888	1.762	1.672	0.233
	CP	0.0678	0.2248	0.2364	0.2582	6.351	1.827	1.755	5.266
0.13	TRUE	0.0676	0.2251	0.2371	/	15.312	4.596	4.363	/
	PCP	0.0676	0.2247	0.2367	0.1219	15.306	4.581	4.347	0.248
	CP	0.0687	0.2225	0.2404	1.3878	16.545	4.417	3.874	7.719
0.25	TRUE	0.0676	0.2251	0.2371	/	29.447	8.839	8.390	/
	PCP	0.0675	0.2247	0.2367	0.1242	29.428	8.805	8.361	0.263
	CP	0.0698	0.2217	0.2448	2.6899	32.710	9.303	10.126	12.341

**Table 7:** The results of modal assurance criterion in closely spaced modes cases in free vibration.

α	Method	Modal Assurance Criterion (%)		
		1st Mode	2nd Mode	3rd Mode
0.05	PCP	99.99999	99.99999	99.99999
	CP	99.99910	98.99545	99.94721
0.13	PCP	99.99999	99.99999	99.99999
	CP	99.73610	74.28778	96.53389
0.25	PCP	99.99999	99.99999	99.99999
	CP	98.74817	47.74576	58.79833

**Table 8:** Locations and directions of the HCT building test setups.

Setup	Chennel 1	Chennel 2	Chennel 3	Chennel 4	Chennel 5	Chennel 6	Chennel 7	Chennel 8
1	45 N	44 N	44 W	41 N	R40 W	R40 N	-	-
2	33 N	37 N	32 N	32 W	36 W	36 N	R40 W	R40 N
3	25 N	29 N	24 N	24 W	28 W	28 N	R40 W	R40 N
4	17 N	21 N	16 N	16 W	20 W	20 N	R40 W	R40 N

**Table 9:** The results of natural frequencies and damping ratios identified by different methods.

Mode No.	PCP		CP		SSI-COV		SSI-DATA [43]		FDD [44]	
	Freq. (Hz)	Damp. (%)	Freq. (Hz)	Damp. (%)	Freq. (Hz)	Damp. (%)	Freq. (Hz)	Damp. (%)	Freq. (Hz)	Damp. (%)
1	1.230	1.12	1.233	1.58	1.231	1.27	1.243	1.4	1.232	2.17
2	1.289	1.06	1.287	1.51	1.286	1.03	1.290	1.5	1.273	1.97
3	1.454	0.91	1.454	1.07	1.455	0.89	1.457	1.5	1.436	3.35
4	3.864	1.34	3.865	1.33	3.863	1.35	3.868	1.6	3.866	1.44
5	4.247	1.54	4.243	1.13	4.266	1.68	4.415	3.4	4.251	1.67
6	5.336	2.52	5.356	2.52	5.340	2.04	5.386	2.5	5.354	2.06
7	6.365	1.52	6.368	1.49	6.391	1.40	6.445	5.7	6.398	1.60

## Data Availability

Data will be made available on request.
